# The Cellular Choreography of Osteoblast Angiotropism in Bone Development and Homeostasis

**DOI:** 10.3390/ijms22147253

**Published:** 2021-07-06

**Authors:** Georgiana Neag, Melissa Finlay, Amy J. Naylor

**Affiliations:** Rheumatology Research Group, Institute of Inflammation and Ageing, University of Birmingham, Birmingham B15 2TT, UK; GXN648@student.bham.ac.uk (G.N.); MXF965@student.bham.ac.uk (M.F.)

**Keywords:** bone, remodeling, endothelial cell, blood vessel, osteoblast, osteocyte

## Abstract

Interaction between endothelial cells and osteoblasts is essential for bone development and homeostasis. This process is mediated in large part by osteoblast angiotropism, the migration of osteoblasts alongside blood vessels, which is crucial for the homing of osteoblasts to sites of bone formation during embryogenesis and in mature bones during remodeling and repair. Specialized bone endothelial cells that form “type H” capillaries have emerged as key interaction partners of osteoblasts, regulating osteoblast differentiation and maturation and ensuring their migration towards newly forming trabecular bone areas. Recent revolutions in high-resolution imaging methodologies for bone as well as single cell and RNA sequencing technologies have enabled the identification of some of the signaling pathways and molecular interactions that underpin this regulatory relationship. Similarly, the intercellular cross talk between endothelial cells and entombed osteocytes that is essential for bone formation, repair, and maintenance are beginning to be uncovered. This is a relatively new area of research that has, until recently, been hampered by a lack of appropriate analysis tools. Now that these tools are available, greater understanding of the molecular relationships between these key cell types is expected to facilitate identification of new drug targets for diseases of bone formation and remodeling.

## 1. The Function, Anatomy, and Cellular Overview of the Skeletal System

The mammalian skeletal system is highly dynamic with multiple crucial roles, most notably providing architectural and mechanical support to the whole body, protection of the vital organs, metabolic and endocrine modulation, and, in the case of long bones, a site for hematopoiesis [1]. Bones are morphologically compartmentalized into two distinct and contrasting structural arrangements: a cancellous type called trabecular bone and a compact solid type termed cortical bone. Trabecular bone is present within the metaphysis of the long bones, vertebral bodies, ribs, and the iliac crest, while cortical bone forms the outer layer of all bones. Cortical bone bears most of the mechanical load in the body. However, the importance of trabecular bone is more apparent for overall bone strength and resilience during periods of high mechanical force [2,3].

Although the predominant appearance of bone matrix is of a compact mineralised tissue, this unique biomaterial is highly cellular and contains a wide variety of specialised cell types that contribute to its formation, maintenance, and continual remodelling.

Highly regulated processes establish intercellular communication between bone-resident cells, each with its own distinctive role, to ensure a finely tuned balance between bone resorption and bone formation. The major bone-resorbing cell type is the osteoclast. Osteoclasts are multinucleated cells formed by the fusion of mononuclear, monocyte-derived, macrophage precursors, and their main function is resorption of cartilage and bone matrices. Vessel-associated osteoclasts drive cartilage resorption during bone growth [4], whereas bona fide osteoclasts regulate resorption of mineralised bone matrix during bone maintenance and repair. Furthermore, with bone being the largest reservoir for calcium storage in the body, resorption also facilitates calcium release to maintain optimal circulating levels [5]. Osteoblasts play the opposite role to osteoclasts, constituting the major bone-forming cell type. Mesenchymal stem cells (MSCs) are situated at the apex of the osteoblast lineage cell hierarchy, differentiating stepwise into osteoprogenitors, bone lining cells, osteoblasts, and, ultimately, osteocytes [6,7]. Osteoblast lineage cells have numerous roles depending on their differentiation stage. Osteoprogenitors and bone lining cells constitute a pool of immature osteoblasts, maintained in a quiescent state that can be utilized for bone remodelling and repair. Mature osteoblasts ensure production and secretion of a new protein-rich bone matrix and its subsequent mineralization. Osteocytes are terminally differentiated osteoblasts, entombed within mineralised bone, acting as the main regulatory unit of skeletal homeostasis. Additional cells supporting bone homeostasis include endothelial cells (ECs), adipocytes, and chondrocytes.

ECs, in particular, have recently been at the forefront of scientific investigation, due to major technological advances allowing for a detailed exploration of the bone vascular bed; it is now widely accepted that ECs form blood vessels that act as supporting conduits to bone cells. In addition, ECs perform multiple pivotal roles in the vascular system including vasculogenesis, angiogenesis, vascular maturation, and remodelling, as well as control of vascular tone and blood flow [8,9]. In addition to these traditional roles, the bone vascular endothelium mediates the compartmentalization of osteogenesis and hematopoiesis by creating niche local environments favorable to each of these two processes [9,10,11,12,13].

Skeletal development, growth, remodelling, and repair rely on a complex cellular ecosystem, wherein cells are engaged in continuous cross talk via the release of a panoply of autocrine and paracrine signals. This review focuses specifically on the bidirectional molecular interactions that occur between ECs and osteoblast lineage cells and details our current understanding of how these interactions facilitate and control bone homeostasis throughout life.

## 2. Recent Advances in Bone Endothelial Cell Diversity

The vascular system ensures delivery of nutrients and metabolites, oxygen availability, removal of waste products, and trafficking of a wide variety of cells throughout the body using a series of vessels of different sizes and structures, the lining of which is composed of ECs, differentiated from the mesoderm during embryogenesis [14]. The bone vascular network is composed of arteries, the central draining vein, and a myriad of capillaries. The latter form a compact, interconnected vascular bed throughout the length of long bones. In the last decade, three distinct subtypes of blood capillaries have been identified and characterised in the murine bone: types L, H, and E capillaries [10,15,16]. Type H vessels have been subsequently confirmed in the human tibia and femur [17].

A clear distinction between capillary types can be made based on their relative expression of Endomucin (EMCN), a marker for non-arterial vessels, and CD31 (encoded by the PECAM-1 gene), a canonical marker for ECs [18,19]. Types H and E capillaries have higher expression of both EMCN and CD31, whereas type L capillaries express these markers at comparatively lower levels. This highly double-positive expression of EMCN and CD31 is driven by Notch signalling, which prompts the increased transcription of PECAM-1, EMCN, as well as Kinase Insert Domain Receptor (KDR), a Vascular Endothelial Growth Factor (VEGF) receptor [20]. In mice, type L vessels are present during both bone embryogenesis (from embryonic day (E)16.5) and in the postnatal long bone in juveniles and adults (assessed from birth until 70 weeks of age) [10,15,21]. The proportion of ECs with the type L phenotype increases steadily from embryogenesis to maturity (32% to 88.9%, at E16.5 and postnatal day (P)28, respectively) [15]. On the other hand, types E and H vessels present a contrasting trend. The proportion of type E capillaries decreases from E16.5, until nearly undetectable at P28 (58% and 2%, respectively), while that of type H capillaries increases from E16.5 to P6, then decreases until P28 (9%, 26%, and 9%, respectively) [15]. During embryogenesis, the majority of vessels in the murine long bone are types H and E capillaries, whereas postnatally, type L vessels comprise the majority of capillary ECs [15].

**Figure 1:**
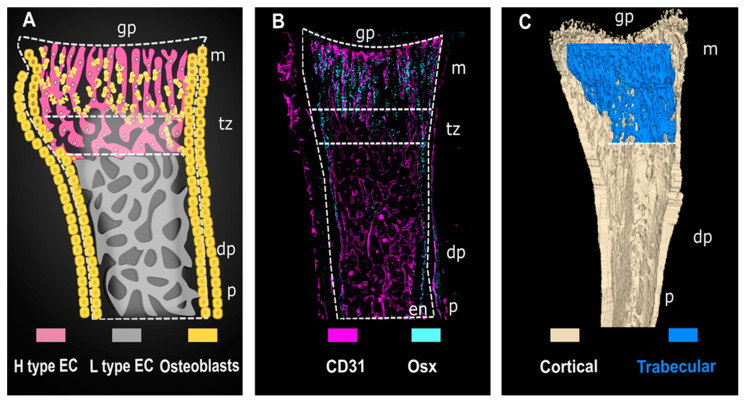
Compartmentalization of the murine long bone and its capillary network. H-type vessels are presented with their main interacting partners, Osx^+^ cells, in the postnatal murine long bone. Left to right: (**A**) Schematic diagram illustrates H- (magenta) and L-type (grey) vessels with their distinct morphology and emphasizes the strict geographical distribution of Osx^+^ osteoprogenitors in the metaphysis as compared to the diaphysis. Osteoprogenitors in the metaphysis (indicated by yellow, irregular-shaped cells) are presented closely associated to H-type capillaries during their migration to sites of trabecular bone formation. Located in the endosteum and periosteum is a pool of osteoprogenitors (indicated by yellow, cuboid-shaped cells) not associated with H-type vessels. H-type vessels are columnar formations perpendicularly oriented against the lowermost point of the avascular growth plate in long bones. L-type vessels are localised to the diaphysis where they form a sinusoidal hypoxic vessel bed surrounded by bone marrow cells (not represented). H- and L-type vessels form a continuous vascular network; the area where these H-type capillaries begin to converge into L-type capillaries has been branded the transition zone, an area with a vascular web of an intermediate phenotype between these two capillaries. (**B**) High-resolution confocal imaging of CD31 and Osx, labelling endothelial cells (magenta) and osteoprogenitors (cyan), respectively. Postnatal day 4 (P4) tibia sectioned on the axial plane illustrates the specific localization of bone-forming cells around H vessels in the metaphysis, endosteum, and periosteum. Note the formation of arches and bulges at the caudal point of the growth plate, as previously described. (**C**) Illustration of the compartmentalization of the long bone. Pseudocolored, micro-computed tomography, 2D plane snapshot from a 3D bone mesh model. Trabecular bone (blue) is superimposed on cortical bone (ivory). The uppermost and lowermost regions of the long bone start and end at the epiphysis at each end of the bone (not shown). The growth plate is localised at the interface between the epiphysis, and the metaphysis, the region where trabecular bone is localised in the long bone (blue). The middle part of the bone, the diaphysis, is void of trabecular bone and houses the bone marrow. Abbreviations: diaphysis-dp, endosteum-en, growth plate-gp, metaphysis-m, periosteum-p, H-type vessel to L-type vessel transition zone-tz.

Invasion of the vascular plexus into the long bone occurs between E15.5 and E16.5, succeeding initiation of MSC osteoblast differentiation at E14.5 and preceding initiation of bone formation at the primary ossification center [15,22]. At birth, type H vessels are expressed throughout the long-bone vascular bed; however, postnatally their geographical distribution becomes more distinct and they are located almost exclusively within the metaphysis region. Types H and L vessels in the postnatal long bone can be readily distinguished according to their localization and morphological organization (illustrated in Figure 1) [15]. Type H vessels are columnar capillaries that end in anastomosis bulges at the caudal point of the avascular growth plate [10]. Recently published work by Romeo and colleagues identified an important role for the anastomosis bulges in angiogenesis during bone growth, particularly during resorption of the chondrocyte-deposited matrix in the growth plate region [4]. Conversely, type L vessels are confined to the diaphysis, where they form a sinusoidal, highly branched, and relatively hypoxic vessel bed, through which immune cells can readily pass from the bone marrow into the bloodstream [16].

Lineage-tracing experiments have confirmed that type E ECs are developmentally the most upstream capillary EC in the bone, constituting the origin of types L and H ECs [15]. Type H vessels have been identified in the long bones (murine and human) and alveolar bones (murine), as well as the spine, sternum, and calvarium (murine) [10,23,24]. In the murine long bone, peak vessel density is reached at P6. However, this progressively declines during adulthood and aging [15,21]. A similar decline in type H vessel number and density has also been reported in humans with increasing age, a loss that has been associated with osteoporosis and osteopenia, although a causal link in humans has yet to be established [17,25].

While type L capillaries are surrounded by hematopoietic stem cells, type H vessels interact with osteoblasts [15,26]. A strong indicator that type H vessels are essential for the coupling of osteogenesis and angiogenesis is demonstrated by the fact that both type H and E capillaries are the only ones surrounded by osteoprogenitor cells in the whole bone, with the exception of transcortical vessels in the endosteum and periosteum [10,15,21], although it remains unclear whether the newly identified transcortical blood vessels [27,28] are in essence type H capillaries.

## 3. Bidirectional Cross Talk between Endothelial Cell and Osteoblast Regulates Bone Formation during Embryogenesis and Development

Embryonic osteogenesis occurs through developmental processes of either endochondral or intramembranous ossification. Long bones are formed via endochondral ossification, a process during which the pre-existing matrix of an avascular, cartilaginous, and relatively homogenous tissue template (anlage) is resorbed and remodelled into a highly vascularised, architecturally complex, multicellular, and multifunctional bone tissue. Remodelling of the initial anlage is accomplished via osteoclast degradation and co-invasion of vessels and osteoblast progenitor cells differentiated from MSCs. Osteoblast lineage commitment unfolds in the perichondrium of the anlage where MSCs undergo stepwise differentiation into osteoprogenitors and other cell types. Consequently, osteoprogenitors undergo proliferation and proceed to build the cortical bone collar (perichondrium) that surrounds the anlage [22,29]. On the other hand, flat bones, such as the calvarial bones of the skull, form through intramembranous ossification with no initial cartilaginous template required. This process relies on local MSCs to undergo a stepwise differentiation into osteoprogenitors and then into osteoid-secreting mature osteoblasts. Both of these developmental processes have been reviewed in depth previously [30,31,32].

The first stage of endochondral bone formation during embryogenesis is initiated by MSC differentiation. Stem cells destined to differentiate into osteoblasts will progress through three precisely timed and regulated stages of differentiation, each of which can be identified by changes in the expression of a well-defined set of proteins and transcription factors. This has been reviewed in more detail previously [6,33,34], and can be summarised as follows.

Commitment to the lineage: Under the influence of the local niche MSCs begin to selectively express osteoblast master transcriptional regulators: Runt related 2 (RUNX2), β-catenin, and Osterix (OSX). The concerted actions of these regulators and of locally expressed stem cell factors induce osteo-lineage commitment programming, giving rise to cells interchangeably termed progenitor cells, osteoprogenitors, immature osteoblasts, and pre-osteoblasts, herein called osteoprogenitors.

Mitosis: Following commitment, osteoprogenitors, marked by OSX but lacking Collagen type I expression, undergo a period of high proliferation.

Osteoid production and mineralization: Cuboidal-shaped mature osteoblasts, characterised by production of collagen I and alkaline phosphatase, produce the extracellular matrix of the bone that later undergoes mineralization.

Given the plethora of functions accomplished by osteoblast lineage cells throughout their differentiation journey, it comes as no surprise that cellular metabolic functions differ between cell differentiation status, in line with geographical oxygen and nutrient availability, both of which are locally delivered by vessels. Osteoblast differentiation requires increased oxygen consumption, as previously reported in vitro [35], in silico, and in vivo [36]. Low oxygen availability hampers osteogenesis commitment by limiting the MSC metabolic shift towards mitochondrial oxidative phosphorylation, a change driven by the increased expression levels of the oxygen-sensing transcription factor, Hypoxia-Inducible Factor 1 Alpha (HIF-1α) [37,38]. Hypoxia and HIF-1α also impede osteoblast proliferation via the coupled HIF-1α-Osterix signalling pathway, which inhibits WNT signalling [39,40]. While MSCs are highly dependent on oxygen availability during their differentiation and proliferation, they exhibit multivalency in relation to nutrient availability, being able to either undertake glycolysis, fatty acid oxidation, or ketolysis (ketone body oxidation) to meet their metabolic needs (reviewed in detail previously) [41,42]. On the other hand, substrate excess, such as high-glucose environments associated with diabetes, can decrease osteoblast migration, due to a decline in phosphorylation of cell motility regulators protein kinase B (AKT) and mitogen activated protein kinase ERK [43]. This body of work demonstrates the importance of bone vascularization for osteoblast regulation through oxygen and nutrient availability; however, there is still a lack of clarity regarding the involvement of EC-derived signalling in this process.

**Figure 2:**
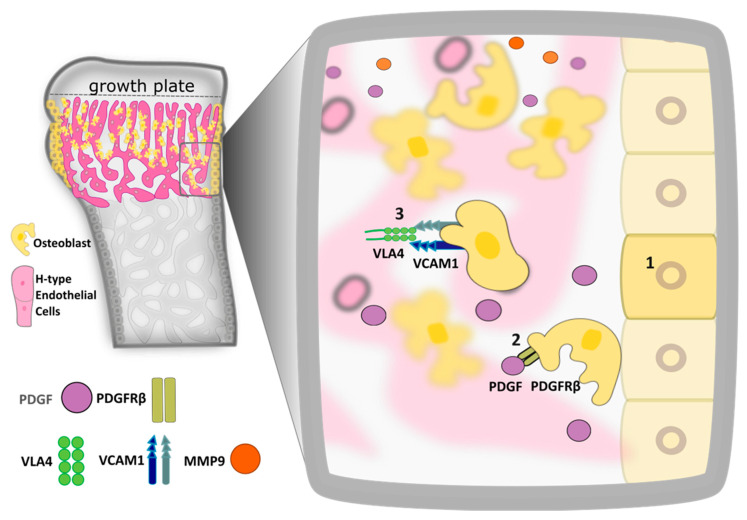
Cellular and molecular factors regulating osteoblast-EC angiotropism during bone growth and repair. Diagram illustrates the three main stages of osteoblast angiotropism. (1) MSC to osteoprogenitor differentiation occurs in response to local cues from the bone niche. Factors include oxygen and nutrient availability as well as HIF-1α and WNT/β Catenin signaling. Note the different morphology of osteoprogenitors not associated to vessels and their localization in the endosteum and periosteum. (2) Expansion of the Osx+ osteoprogenitor pool via proliferation under the influence of PDGF-PDGFRβ signaling, which favors maintenance of a pro-migratory immature osteoblast phenotype. (3) Osteoblast migration to sites of bone formation and repair alongside expanding H-type capillaries. PDGF acts as a chemoattractant to PDGFRβ^+^ osteoprogenitors in the near vicinity. Subsequent to PDGF-PDGFRβ signaling, MMP-9 is also secreted by osteoblasts to aid in degradation of the surrounding matrix. MMP-9 expression is particularly increased at the vascular front during active angiogenesis. Cell-to-cell contact is required to facilitate osteoblast adhesion to EC. For example, VLA4-VCAM-1 interaction facilitates osteoblast angiotropism by enabling anchorage of the osteoblast cell membrane to H-type vessels.

The second stage of endochondral bone formation, whereby the remaining cartilaginous template is replaced with bone tissue, is initiated in embryogenesis and continues postnatally. In embryonic life, this starts with the invasion of types E and H ECs into the primary ossification center [15], followed by a subsequent longitudinal and radial expansion of vessels, and then terminates at the secondary ossification centers. Vascularization of the bone template spans from E15.5 until peak bone mass is attained, which, in mice, is at 11–12 weeks of age [10,15,44]. Expansion of the bone vascular supply relies heavily on sprouting angiogenesis [45,46], a sequential process involving: (1) initial selection of EC tip cells, which subsequently initiate and lead the vessel sprout; (2) EC migration for extension of the sprout and vessel tube formation; (3) connection of vessel sprouts via anastomosis and sprout pruning [47,48,49,50,51]. During the angiogenesis-driven expansion of the capillary network, type H vessels and osteoblasts engage in a feed forward loop and trigger the secretion of a plethora of angiocrine prototypical signals [15,52] (Table 1). Type H capillaries also act as guides, supporting OSX^+^ osteoprogenitors bound to them and driving their migration to sites of bone formation, a process known as angiotropism [22,42]. This is better defined as the propensity of cells to have an affinity for and move towards and along blood vessels, a process that was first described in the 1980s in lymphoma migration [53]. EC-mediated osteoblast angiotropism on the outside of the vessel lumen has been identified more recently [22]. However, the fundamentals of this process and its molecular mechanisms have yet to be fully characterised. During osteoblast angiotropism, OSX^+^ cells behave like pericytes, extending their cellular processes and wrapping themselves around the invading blood vessels to facilitate migration towards the bone metaphysis, where they proceed to form trabecular bone [12,22] (Figure 2).

Osteoblast angiotropism is regulated by interactions with ECs, which are restricted to types H and E vessels in the metaphysis, the H- and E- type EC specific interaction with OSX^+^ cells was demonstrated with the aid of EC-Osteoblast co-cultures [10,15]. In vitro co-culture of H or E type ECs with MSCs demonstrated that either EC type induced morphological changes in 3D spheroids. Specifically, MSCs underwent osteo-lineage commitment in the presence of ECs, as confirmed by increased expression of RUNX2, Osteocalcin, and Alkaline Phosphatase (ALPL). Over time, osteoblasts also migrated to the edges of the spheroid, whereas EC condensations formed in the center [15]. While this demonstrates the importance of cell-to-cell contact for osteoblast angiotropism, previous work by Clarkin and colleagues indicated that it is not required for osteoblast differentiation by EC, thus beautifully showcasing the importance of EC-secreted factors in driving osteoblast differentiation [54]. In these experiments, non-contact co-culture of human umbilical vein endothelial cells (HUVEC) and human osteoblasts also resulted in increased osteoblast differentiation and a sustained rise in secretion of ALPL from osteoblasts [54].

The blood flow in types H and E capillaries, characterised by high shear rates, is probably another key factor promoting osteoblast angiotropism. Migratory osteoblasts exhibit a preference for higher shear stress, which positively correlates with regulation of flow directionality [55]. Modelling of low blood pressure through pharmaceutical interventions or artery ligation, which is de facto accompanied by low shear rates and stress, results in decreased density of OSX^+^ cells in the metaphysis and a loss of bone formation capacity as measured by micro-computed trabecular bone parameters [11]. From an anatomical perspective, the reported higher shear rates linked to high blood velocity in types H and E vessels (compared to sinusoidal type L capillaries in the diaphysis) can be explained by their direct connection to arteries and arterioles, which also grants them a highly oxygenated status [11]. Elevated oxygen concentrations (compared to type L capillaries) allow them to meet the greater energy demands of bone formation in the metaphyseal region, which is the site of trabecular bone formation. This is in accordance with the high turnover rate of trabecular bone, making this area more metabolically active than the bone cortex [56].

A seminal study by Langen and colleagues identified that changes in the expression of EC adhesion molecules, such as the ones described by the EC-specific genetic deletion of Integrin β-1 (ITGB1), can also affect osteoblast differentiation and, potentially, angiotropism [15]. ITGB1 is a multifaceted integrin that is the component of various integrin complexes including Very Late Antigen 4 (VLA4), a protein with an ascribed role in cell adhesion [57]. EC-specific genetic deletion of ITGB1 leads to transformation of type H vasculature morphology, function, and metabolism. Namely, type H vessel columns are shorter, more branched, and lose their specific parallel organization. This is accompanied by a sustained reduction in proliferation and a decrease in the non-hypoxic area characteristic of the metaphysis where type H vessels reside. Changes in the metabolic function of these ITGB1-deficient ECs are also driven by a decrease in the activity of the phosphorylated mitogen activated protein kinase ERK (phospho-ERK1/2) [15]. These type H vessel changes coincide with defective bone formation, as exemplified by shorter bones with reduced trabecular bone parameters. Perturbed bone formation in this instance is characterised by improper osteoblast differentiation and maturation, with mutant mice displaying increased density of RUNX2-positive cells and decreased OSX^+^ cell abundance as well as a diminished deposition of proteins secreted by mature osteoblasts or osteocytes (Collagen type I and Osteocalcin). Additionally, considering the reported bone shortening alongside contraction of the type H capillary columnar front in ITGB1 knockout mice, abnormal osteoblast angiotropism is strongly suspected [15]. The same study reported similar but milder observations following genetic deletion of genes coding for the extracellular matrix (ECM) proteins, LAMA5 and Osteopontin, suggesting that ECM proteins are key drivers of osteoblast maturation and vascular angiogenesis, an area that warrants further investigation.

In a reversal of roles, osteoblasts, osteoclasts, and hypertrophic chondrocytes also secrete proangiogenic factors to support angiogenic processes [10,45,46]. For instance, VEGF is not only secreted by ECs as an autocrine signal, it is also released from osteoblasts [58]. This process is triggered by Fibroblast Growth Factors, hormones, HIF-1α, and other signals secreted in the bone niche that induce secretion of lipid-signalling mediators such as Prostaglandins (e.g., PGE2 and PGI2) from ECs, which prompt VEGF secretion from osteoblasts [54,59,60].

The bidirectional nature of the EC–osteoblast relationship is further evidenced by research by Xu and colleagues, who showed that in addition to VEGF, osteoblasts secrete other proangiogenic factors that regulate and increase type H vessel density [61]. In particular, they identified osteoblast-derived SLIT3, which interacts with VEGF signalling mediator, ROBO1, expressed on ECs, to positively regulate both endothelium expansion and bone formation. The potent role of SLIT3 was evidenced during bone growth, where genetic deletion of SLIT3 in osteoblasts results in a decrease in cortical thickness and a reduction in trabecular bone volume.

## 4. Osteoblast Differentiation and Angiotropism Control Bone Healing in Mature Tissues

Mature bone is continually undergoing renewal and micro-damage repair [62,63]. This dynamic property of bone is attained through remodelling, whereby bone is resorbed then new bone matrix is deposited accordingly, to maintain and meet ever-changing physiological demands throughout an individual’s lifetime. This process requires coordination and appropriate balance between the rates of resorption and formation, which have been reviewed extensively [64,65]. Activation is the first distinct phase of bone remodelling, where a Bone-Modelling Unit (BMU) forms at the site to be remodelled and consists of precursor and differentiated bone cells, connective tissue, and blood vessels [62,63].

In the case of injury or trauma adequate local vascularization of the assembled BMU, is vital for successful repair and regeneration of damaged bone. ECs aid in the recruitment and differentiation of osteoblast progenitors through similar, if not identical, mechanisms as during embryogenesis [22]. Fracture healing is characterised initially by formation of a fibrin clot, which is highly vascularised and rich in VEGF. It is believed that this consistently high concentration of VEGF, initially produced by fibrin clot platelets and thrombocytes, and later secreted by osteoblast lineage cells via HIF-1α activity, aids in differentiation of osteoblasts and initial angiogenesis and osteogenesis [66,67,68]. Successful remodelling of the initial fibrin clot into bone tissue recapitulates many of the processes seen during endochondral ossification [69]. Therefore, fracture and osteotomy studies are used to study both healing and regenerative processes [70,71,72].

Long bone remodelling and repair relies on activity of OSX^+^ osteoprogenitors originating from a pool of skeletal stem progenitor cells spread across different locations in the long bone including the periosteum [22,73] and the bone marrow [74,75]. Osteoprogenitors, which are additionally marked by expression of Cathepsin K (CTSK) [73], are located to the periosteum, whereas Leptin receptor (LEPR) and C-X-C motif chemokine ligand 12 (CXCL12) double positive cells reside in the bone marrow [74,76]. Following bone injury, these skeletal stem progenitor cells give rise to OSX^+^ osteoprogenitors, which undergo a period of intense proliferation [77], followed by migration to newly forming bone areas by association with vessels [22]. Recent studies provide evidence that geographical location within the bone can modulate lineage commitment potential, most likely due to the direct influence of signalling of cells in the near vicinity [22,74,76]. CXCL12^+^ LEPR^+^ progenitors are localised in close proximity to L capillaries or arterioles in the bone marrow and contribute to osteoblast and osteocyte generation following bone injury, dependent on WNT-β Catenin signalling [76]. All these osteoprogenitors have been only shown to regenerate cortical bone [73,76]. However, the fracture models employed failed to address their potential to regenerate trabecular bone, a phenomenon that remains to be elucidated. It also remains unclear how differentiation and maturation of osteoprogenitors is regulated by EC-derived signals. While the transcriptomic signature of osteo-lineage cells is becoming more clearly delineated, there is still little insight into the metabolic activity of MSCs whilst undergoing differentiation into osteoprogenitors during fracture healing and regulation of these processes by ECs.

One of the other mainstay processes for fracture healing is vessel invasion into the fracture callus. Newly formed capillaries enable a sufficient supply of adequate nutrition, oxygen, and systemic factors and facilitate the recruitment of osteoclast and osteoblast progenitor cells to sites of bone repair [42,78]. The importance of vascularization of the fracture callus for successful bone healing has been known for over 3 decades [79] and has been well described previously [58,80] and recently [26,42]. Failure of clot formation, or its inadequate vascularization, is a hallmark of fracture non-union, where fractures fail to heal adequately [81]. Inhibition of angiogenesis via physical barriers, or by blocking VEGF signalling, are widely used experimental techniques to probe the importance of vascularization for bone formation during fracture healing. Angiogenesis blockade results in a sustained decrease of osteoblast and osteoclast migration towards sites of bone formation [82,83,84,85] and, in line with this, Van Gastel and colleagues utilised an in vivo periosteal bone healing model, employing polycarbonate filters, to inhibit revascularization of a periosteal callus implanted in a femur fracture [42]. This resulted in a reduction in the overall cell numbers populating the bone callus area, alongside a decrease in cell proliferation, and an increase in cell death, similarly to results presented in previously published work [82,83,84,85].

To further expand our understanding of the role of vascularization in osteo-lineage commitment of skeletal progenitors, Van Gastel et al. reported that reduced vascularization of the callus was characterised by an expansion of cells expressing the chondrocyte commitment master regulator, SOX9, indicating a preferential chondrocyte lineage commitment at the expense of osteo-lineage differentiation [42]. The observed changes could be explained by the importance of lipid availability for MSC to osteoblast differentiation considering that lipid scarcity results in chondrocyte commitment [42]. Delving into this further, the role of angiogenesis progression for the composition of the periosteal callus proximal to the fracture site was predicted using computational modelling and then tested in vivo and demonstrated the importance of vascularization for reduction of the hypoxic environment [36]. An increase in callus vascularization led to changes in MSC fate, promoting osteoblast lineage commitment to the detriment of differentiation into chondrocytes, resulting in a faster rate of healing [36].

Osteoblasts regulate expansion of the endothelial vessel front as well as their own invasiveness via release of the extracellular matrix degradation protease, Matrix Metallo-Proteinase 9 (MMP-9) [4,26]. Increased MMP-9 production by skeletal stem and progenitor cells is a downstream response to (Platelet Derived Growth Factor and Platelet Derived Growth Factor Receptor β) PDGF-PDGFRβ signalling [26]. Using distinct profiles as a guide to osteoblast differentiation stages has allowed dissection of the paracrine signalling of OSX^+^ immature versus Collagen I-secreting mature osteoblasts at the molecular level. OSX^+^ osteoprogenitors produce a plethora of angiogenic and proliferative factors when compared against whole long bone or calvaria digestion without enrichment for OSX expression. Specifically, qPCR analysis of the OSX^+^ cell transcriptome showed increased expression of VEGF, Angiopoietin-1, and PDGF receptor β mRNA [22,86]. The well-characterised cellular migration and invasiveness regulator, SLIT3 is a newly identified factor which has been shown to also expand the endothelial footprint in the bone [87,88]. SLIT3 is another osteoblast-derived protein that mediates bone healing by directly acting on ECs to drive callus vascularization. This was confirmed following local administration of recombinant SLIT3 in a model of open femoral midshaft fracture healing. Treatment with SLIT3 resulted in thicker cortical bones, a rise in trabecular bone volume and stiffer bones with a higher load-bearing capacity [61].

The expression of the aforementioned angiogenic factors is a clear indication of cross talk occurring between ECs and osteoblasts and provides evidence of their bidirectional communication and of a mechanism by which vessel and osteoprogenitor invasion is coupled [22,86]. Angiogenesis and osteogenesis coupling plays a key role in bone healing. However, identification of the precise vessel type invading the callus and its spatio-temporal and molecular regulation remains elusive, even if H-type vessels are strongly suspected to be the blood vessel type regulating bone healing, this is yet to be tested experimentally. Van Gastel et al. have nonetheless shown that bone repair is reliant on blood vessel expansion into the fractured zone [42]. Evidence has mounted of the importance of the vasculature in regulating mature bone remodelling whereby newly forming capillaries influence osteoprogenitor cell fate by supporting their angiotropism to sites of bone remodelling and repair [22,26] (Figure 2). EC-secreted PDGFB binds PDGFR-β expressed on migrating osteoblasts, maintaining them in an immature state and upregulating their intensely proliferative status. PDGF downstream signalling also leads to expression of cell adhesion molecule VCAM1 by osteoblasts to presumably facilitate their migration on EC via interaction with Integrin β1 Integrin α 4 dimer VLA4 [26].

During the maturation process osteoblasts lose their attachment to ECs, as shown by Maes et al. [22], further indicating that cell-to-cell contact between ECs and osteoprogenitors regulates maintenance of osteoblasts in an immature and highly proliferative state, favorable to angiotropism [46]. Mature osteoblasts adhere to the cartilage and work as functional units, via a series of tight, gap, and adherens’ junctions, to produce and lay down the new osteoid, a protein-rich bone matrix [22,26]. The newly deposited osteoid is then mineralised through nucleation of hydroxyapatite to collagen fibrils, which are initially regulated by the non-collagenous proteins, such as Osteocalcin and Bone Sialoprotein, found within this immature bone matrix [89]

## 5. The Extent of Osteocyte-Endothelial Cell Cross Talk Remains an Important, and Outstanding, Area for Research

The importance of the cross talk between osteoblast lineage cells and ECs implies that such a relationship will continue as osteoblasts further differentiate into the most numerous bone cell type at skeletal maturity, osteocytes. Given the vital roles osteocytes play in preserving optimal bone health, investigating whether such an association exists, and how it may or may not contribute to the repair and maintenance of mature bone, is entirely justified.

Following osteoid deposition, around 10–30% of the osteoblast population differentiates into osteocytes [90,91]. Osteocytes are long-lived, terminally differentiated osteoblasts and estimates calculate that the average osteocyte lifespan is in excess of 10 years. Osteocytes use their highly dendritic morphology to create a vast, interconnected cytoplasmic network throughout the bone, via gap junctions at dendritic contact points. This enables communication between osteocytes within the network, as well as with other bone cells, blood vessels, and the inner and outer surfaces of bone, through chemical and mechanical signalling transduction [92]. It has been shown that physical connections are present between osteocyte dendritic processes and blood vessels, where the dendrites are oriented in the direction of the blood supply [93,94]. The highly extensive cytoplasmic lacunocanalicular network enables inter- and extra-cellular communication through chemical and mechanotransduction signalling. Thus, this physical connection suggests that the relationship between osteocytes and ECs has a communicative function.

Investigations have found that the cross talk between osteocytes and ECs has roles in regulating angiogenesis. Culturing of HUVECs in osteocyte-conditioned media results in an increase in expression of genes with a proliferative phenotype, including PDGFR and MMP-9, which is closely followed by an increase in HUVEC proliferation, migration, and formation of capillary-like vessels [94]. In addition, the same study indicates that osteocyte-expressed VEGF induces MAPK–ERK phosphorylation in HUVECs, a signalling pathway required to promote EC proliferation in the earlier stages of angiogenesis [94].

Additional work also identifies an important role in regulation of angiogenesis and bone repair for apoptotic osteocytes. When bone experiences trauma, osteoclast progenitor cells are activated by the apoptotic bodies of newly exposed apoptotic osteocytes through stimulation with Tumor Necrosis Factor- α (TNF-α), a known driver of osteoclastogenesis, independent of Receptor Activator of Nuclear factor Κappa-β (RANK)-Receptor Activator of Nuclear factor Κappa-β Ligand (RANKL) activity [95] In addition, apoptotic osteocytes also aid in the delivery and recruitment of osteoclast progenitors by regulating angiogenesis at the damaged site and adhesion of circulating osteoclast progenitors to the vascular endothelium [96]. There is an upregulation of VEGF production by apoptotic osteocytes, which encourages EC proliferation and tubule formation, as well as branching at the site of damage [97,98]. Furthermore, there is an increased expression of Interleukin-6 (IL-6) and soluble Interleukin-6 Receptor (sIL-6R) by apoptotic osteocytes and TNF-α by osteoclast progenitors. This precedes endothelial ICAM-1 expression [95,99]. As a result, IL-6, sIL-6R, and TNF-α increase the adhesion of circulating osteoclast progenitors to the vascular endothelium via ICAM-1 [100], which promotes localised recruitment and activation of these progenitor cells to osteoclasts [101]. However, excessive osteocyte apoptosis can result in abnormal angiogenesis, which is implicated in imbalanced bone remodelling, resulting in deleterious bone conditions such as osteoporosis. Recently, it has been discovered that osteocyte apoptosis also occurs in response to the accumulation of Advanced Glycation End products (AGEs) [102]. AGEs result from proteins and lipids that have been non-enzymatically modified and their accumulation is a common occurrence in metabolic conditions, such as type II diabetes, which also comes hand in hand with higher bone fracture risks [103,104]. Chen et al. described a mechanism whereby incubation of the osteocytic cell line, MLO-Y4, with AGEs induced osteocyte apoptosis and, at the same time, upregulated the expression of proangiogenic factors, IL-6 and VEGF-A [102]. It can be assumed that, in vivo, a subsequent angiogenic effect would occur and result in significant inflammation and increased osteoclast activation. Increased osteoclast activation then further implies increased bone resorption and bone destruction, thus accounting for the higher incidence of fractures, osteopenia, and osteoporosis that occur in type II diabetic patients which readily accumulate AGEs [105]. Growing evidence is enabling the recognition that there is a strong correlation between AGE accumulation and increased bone fragility [106,107,108]. However, the exact mechanism for this has not been conclusively established and requires further investigation.

Sclerostin (SOST), a glycoprotein primarily expressed by osteocytes, is an inhibitor of bone formation and was more recently discovered to also regulate angiogenesis. It was previously found that its expression increases EC migration [109], leading to the discovery that sclerostin can promote angiogenesis by increasing production of proangiogenic factors, VEGF and Placental Growth Factor (PLGF) [96]. Furthermore, the same publication also found SOST can also directly induce angiogenic effects by binding to its receptor, low-density lipoprotein receptor-related protein 6, expressed on HUVECs in response to SOST exposure and subsequently antagonising WNT signalling [96].

A common theme among the studies investigating osteocyte-EC cross talk identifies the need for an appropriate model in which to study primary osteocytes, rather than a cell line, to validate initial findings. The location of osteocytes within bone has posed the main barrier to enabling further investigation of their physiological roles. Furthermore, removal of osteocytes from the bone and into culture causes them to rapidly dedifferentiate back into an osteoblast- or fibroblast-like phenotype, making them extremely challenging to study in vitro [110]. Several cell lines have been produced to study the osteocyte at different differentiation stages, with MLO-Y4 being the most commonly used as it represents a mature stage osteocyte. However, their low expression of SOST, in comparison to physiological levels, and the requirement of exogenous chemicals, such as Thiazolidinediones, makes them an imperfect cell line for in vitro modelling [111,112]. Indeed, the study of any cells cultured as a 2D monolayer is unlikely to ever adequately reflect the environment found in vivo, and this is particularly true of osteocytes whose extracellular environment is so intrinsically linked to their cell activity and behavior. The comparison of 2D and 3D cell culture has been recently reviewed [113], concluding that the advantages intrinsic to 3D models will inevitably change the way in which research and discovery is conducted. Over the last 5 years, a number of long-term osteocyte culture methods have been developed [114,115], which could begin to enable the dissection of some of these unanswered research questions and hypotheses, including elucidating the details of the signalling relationship between osteocytes and ECs. This further investigation may yet reveal further vital roles played by osteocytes and bridge the gaps in our knowledge of the link between angiogenesis and osteogenesis during bone development, growth, repair, and maintenance, with implications for treatment or prevention of detrimental bone conditions.

## 6. Conclusions

Endothelial cells and osteoblasts engage in a continuous and complex courtship ritual from embryogenesis, throughout postnatal life and in aging. Blood vessels are integral and crucial to the differentiation of osteoblasts by influencing MSC differentiation into various osteoprogenitor subtypes, reflecting the different bone tissues and their local niche environments. During bone growth and bone maintenance throughout maturity, endothelial cells act as companions to osteoblasts, guiding them to sites where osteogenesis is necessary, providing them with proliferation signals and supporting their angiotropism by expressing molecules that directly interact with the osteoblasts to keep them bound to EC and supplying glucose and fatty acids for their metabolic needs. In aging, the remarkable decline in vessel density characteristic of age-associated alterations [116] is also marked by a sustained change in type H capillary morphology and, presumably, function, this is swiftly accompanied by a significant decrease in the number of osteoblasts in the bone metaphysis [21]. This impairment in osteogenic potential can leave bones porous, brittle, and prone to fracture. However, the ever-increasing understanding of the cellular interactions that control these processes, combined with rapid improvements in in vivo/ex vivo and intravital imaging and in vitro 3D culture methods, will undoubtedly lead to the identification of novel signalling pathways controlling endothelial cell–osteolineage cell cross talk and ultimately new treatment methods for bone diseases.

## Figures and Tables

**Table 1:** Pro-angiogenic factors expressed by type H EC within the developing and growing bone [15]. A brief overview of their known function is given.

Protein	Role
NOTCH4 SEMA6	Angiogenesis-regulators of stalk/tip cell phenotype balance
FLT1 FLT4	Angiogenesis-VEGF receptors
Roundabout family receptor 1 (ROBO1)	Angiogenesis-VEGF signaling mediator
Claudin5 (CLD5)	Vascular permeability-cell-motility and vascular permeability mediator EC tight junction protein
